# Pediatric sepsis prediction: Human in the loop framework

**DOI:** 10.1371/journal.pdig.0001045

**Published:** 2025-11-04

**Authors:** Radha Nagarajan, Sandip A. Godambe, Raina Paul, Ryan Tennant, Kanwaljeet J. S. Anand, Emma Sandhu, Nicole Abrahamson, David Gibbs, Charles Golden, Leo Anthony Celi, Steven Martel

**Affiliations:** 1 Rady Children’s Health, Orange, California, United States of America; 2 University of Waterloo, Waterloo, Ontario, Canada; 3 Stanford University, Palo Alto, California, United States of America; 4 Massachusetts Institute of Technology, Cambridge, Massachusetts, United States of America; Liverpool John Moores University - City Campus: Liverpool John Moores University, UNITED KINGDOM OF GREAT BRITAIN AND NORTHERN IRELAND

“*If you can look into the seeds of time and say which grain will grow and which will not, speak, then, to me” Act I, Scene 3, Macbeth, William Shakespeare, 1606–1607.*

Pediatric sepsis remains one of the most significant threats to global child health, contributing to substantial mortality, long-term morbidity, and sequela that extend far beyond the initial illness with significant economic burden. Sepsis in neonates and children under 19 arises from a dysregulated host immune response to microbial burden (e.g., bacterial, viral, fungal, parasitic), contributing to approximately half of the 50 million global sepsis cases annually [1]. Diagnosis of pediatric sepsis has been acknowledged as challenging, accompanied by rapid progression to septic shock, and abrupt onset of hemodynamic collapse [2]. *Pediatric Sepsis Prediction* (**PSP**) using machine learning (ML) and artificial intelligence (AI) approaches [3–5], has emerged as an important clinical goal, Fig 1. This perspective outlines fundamental challenges and presents a compelling case for an augmented *human-in-the-loop* (**HITL**) framework for PSP. The proposed HITL framework encourages the concerted working of AI and ML models with providers at the bedside within an augmented setting, as opposed to the current practice of generating predictions by models in isolation. HITL also aligns with the broader human factors frameworks such as the Systems Engineering Initiative for Patient Safety (SEIPS) [6].

**Fig 1:**
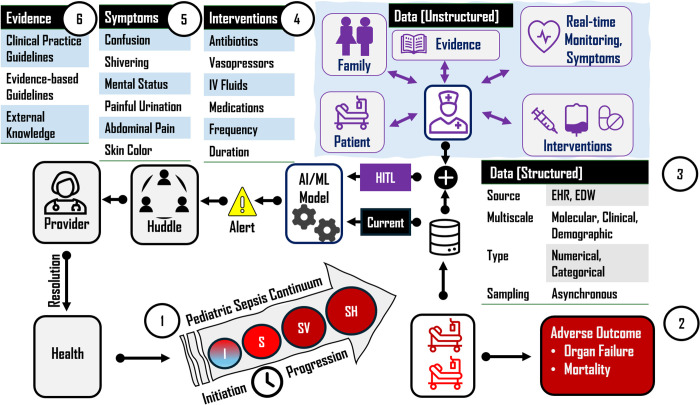
Critical stages in the pediatric sepsis continuum (I, Initiation; S, Sepsis; SV, Severe Sepsis; SH, Septic Shock) and adverse outcomes is shown by (1) and (2), respectively. Current pediatric sepsis alert generation using structured data elements (3) is represented by the black box (Current), and the proposed augmented human-in-the-loop (HITL) framework using multimodal data and concerted working of providers with structured (3) and unstructured data elements (4), (5), (6) is shown by the blue box.

Standardized scoring criteria such as systemic inflammatory response syndrome (SIRS), sequential organ failure assessment (SOFA), and its variants (qSOFA) while used routinely for predicting pediatric sepsis [6] outcomes, rely on adverse events such as organ failure limiting their predictive potential early in the disease progression. Recent efforts have demonstrated the effectiveness of multivariate biomarkers in conjunction with AI and ML approaches such as *single classifiers* (e.g., logistic regression), *ensemble classifiers* (e.g., random forest), and *deep learning* for PSP [3]. However, these approaches predominantly rely on *cross-sectional* profiles and *structured* data elements. More importantly, their failure to accommodate dynamic changes in real-time (e.g., interventions, symptoms, response to treatment), and contemporary decisions at the point of care [7] including the faculty of the provider [8] at the bedside, diminish their predictive potential and generalization ability. Dynamic changes at the bedside is multimodal comprising *structured* as well as *unstructured* data elements [9]. The asynchronous nature of these changes discourages brute-force automation.

Unique challenges accompanying pediatric sepsis prediction are outlined below.

- ***Definition*:** Pediatric sepsis is associated with dysregulated immune responses to infections, hence nonspecific by very definition, making the prediction task challenging. Criteria for defining pediatric sepsis has evolved over time and continue to be debated [10–13].
- ***Predictive Biomarkers*:** Predictive biomarkers widely used for PSP are essentially structured data elements that interrogate the subjects across scales (i.e., *multiscale*) with varying degrees of resolution, and at different time points in the pediatric sepsis continuum, Fig 1. These multivariate biomarkers fall under *molecular*, *clinical*, and *demographic biomarkers*. Predictive potential of molecular biomarkers (e.g., *C-reactive protein*) can be attributed to the fact that disease phenotypes and clinical presentations are preceded by orchestrated changes in the underlying molecular immune signaling mechanisms. However, the molecular signaling cascade accompanying dysregulated immune responses and inflammatory profiles in pediatric sepsis can be nonspecific, impacting their predictive potential. Changes in molecular biomarkers lead to characteristic changes in clinical presentations. Therefore, clinical biomarkers (e.g., *respiratory rate*) can in fact be thought of as the culminating point of the underlying molecular changes. Clinical biomarkers of sepsis can also be nonspecific resembling other serious pediatric illnesses and febrile infections (e.g., *SIRS: systemic inflammatory response syndrome*) limiting their predictive potential. Demographic biomarkers (e.g., *age*), while not actionable, can act as confounders warranting their inclusion in the predictive models.
- ***Asynchronous Sampling*:** Sepsis progression is accompanied by a cascade of events such as tissue damage, organ failure, and mortality. Outcomes that have been of interest from modeling standpoint include *sepsis*, *severe sepsis*, and *septic shock*, with increasing decompensation from sepsis to septic shock, Fig 1. The majority of current PSP models have focused on predicting sepsis from cross-sectional profiles [3], where the data is sampled asynchronously across the subjects in a given time window. Since the patients can be at different points in the disease continuum with varying rapidity of the disease progression, cross-sectional profiling is susceptible to inflated variance impacting the model performance and generalization ability. While longitudinal profiling can ameliorate some of these challenges, the timing and frequency of administering interventions (e.g., IV fluids) can exhibit marked variations between the subjects. These aspects are especially amplified in the presence of *class imbalance* attributed to relatively low prevalence and sample size of pediatric sepsis subjects (0.05%, 19.38%) [3], especially accentuated in the case of severe sepsis and septic shock.

## Proposed human-in-the-loop (HITL) framework

While several advanced models with varying complexity continue to be explored for PSP, these do not compensate for the failure to accommodate real-time dynamic changes and the faculty of the provider at the bedside. Unlike current models that rely predominantly on cross-sectional multivariate structured data elements, the proposed augmented HITL framework emphasizes the importance of capturing multimodal data comprising structured and unstructured data elements at the bedside in real-time. More importantly, it emphasizes the concerted working of providers and models for enhanced prediction with potential to directly impact trust and enhanced adoption of these models in clinical workflows. Asynchronous changes at the bedside can vary across subjects and may include timely intravenous fluids and antibiotic medications at the bedside reducing kidney and neurological sequelae altering the patient’s disease trajectory. Symptoms (e.g., mental status) observed by a provider in communication with the patient and family may not be readily apparent in the retrospective structured data elements used to train current PSP models, Fig 1. Since not all interventions, symptoms, and dynamic changes at the bedside may be relevant, Fig 1, brute-force automation is not an option. The HITL framework overcomes this challenge by leveraging the faculty of providers for identifying qualifying changes for inclusion as potential predictors. The proposed HITL framework addresses critical gaps in PSP and is expected to impact outcomes favorably.
